# Risk Factors for the Development of Hemophagocytic Lymphohistiocytosis in Children With Severe Adenovirus Pneumonia: A Single-Center Retrospective Study

**DOI:** 10.3389/fped.2021.654002

**Published:** 2021-10-01

**Authors:** Hua-yong Zhang, Min Xiao, Fan Yan, Mao-rong Zhang, Yong Zhang

**Affiliations:** ^1^Department of Cardiology, Wuhan Children's Hospital/Wuhan Maternal and Child Healthcare Hospital, Tongji Medical College, Huazhong University of Science and Technology, Wuhan, China; ^2^Department of Rheumatology, Wuhan Children's Hospital/Wuhan Maternal and Child Healthcare Hospital, Tongji Medical College, Huazhong University of Science and Technology, Wuhan, China; ^3^Department of Critical Care Medicine, Wuhan Children's Hospital/Wuhan Maternal and Child Healthcare Hospital, Tongji Medical College, Huazhong University of Science and Technology, Wuhan, China; ^4^Department of Respiratory Department, Wuhan Children's Hospital/Wuhan Maternal and Child Healthcare Hospital, Tongji Medical College, Huazhong University of Science and Technology, Wuhan, China

**Keywords:** severe adenovirus pneumonia, hemophagocytic lymphohistiocytosis, risk factor, children, retrospective study

## Abstract

**Objective:** To investigate and analyze the relevant risk factors for hemophagocytic lymphohistiocytosis (HLH) in children with severe adenovirus pneumonia (SAP).

**Methods:** A retrospective study of children with SAP was performed in 30 cases developing HLH and 94 cases not developing HLH from December 2018 to August 2019. The binary logistic regression analysis was used to identify risk factors that were significantly associated with the development of HLH after the univariate analysis, and the receiver operating characteristic (ROC) curve was performed to find out the cut-off value for the significant relevant factors.

**Results:** Two factors were associated with the development of HLH, which were the length of fever (*OR* = 1.331, 95%*CI*: 1.002–1.769) and triglycerides (TG) (*OR* = 17.345, 95%*CI*: 1.358–221.538). The cut-off value of the length of fever was 12.5 days, and the cut-off value of TG was 3.02 mmol/L.

**Conclusion:** Children with SAP who had a duration of fever over 12.5 days and the TG level over 3.02 mmol/L are more likely to develop HLH.

## Introduction

Community-acquired pneumonia (CAP) is one of the most common diseases in childhood (1). Human adenovirus (HAdV), a common pathogen of CAP in children and adolescents, accounts for 5–10% of cases of CAP in East Asia (2, 3). Human adenovirus is a non-enveloped, double-stranded linear DNA virus, which can be classified into more than 60 serotypes divided into seven species (4). Among these, HAdV-3, and HAdV-7 were the most common serotypes causing severe adenovirus pneumonia (SAP) (5). It is reported that about 50% of the HAdV-7-infected cases were diagnosed with SAP, significantly higher than the HAdV-3-infected cases (6). Severe adenovirus pneumonia is characterized by a persistent fever, shortness of breath, wheezing, progressive dyspnea, and multisystem complications. Severe cases of SAP can lead to respiratory failure and death (7).

Hemophagocytic lymphohistiocytosis (HLH) is a rare but life-threatening hematological disorder, which is characterized by prolonged high fever, pancytopenia, hyperlipidemia, hepatosplenomegaly, and hemophagocytic phenomena in bone marrow slides (8, 9). Based on the etiology, acquired HLH is often associated with infection, autoimmune disease, or malignancy. The most common infectious trigger is Epstein-Barr virus (EBV) infection (10). In recent studies, Infection-associated HLH has also been observed in children with SAP (11–14). However, no study has yet reported the risk factors for the development of HLH in children with SAP. Therefore, this study summarized the clinical data of 124 SAP cases, aiming to find out the risk factors for the development of HLH in children with SAP.

## Materials and Methods

### Study Design and Participants

A retrospective observational study was performed. All 124 individuals with SAP enrolled in this study were identified between December 2018 and August 2019 at Wuhan Children's Hospital/Wuhan Maternal and Child Healthcare Hospital, Tongji Medical College, Huazhong University of Science & Technology. The clinical features of SAP-related HLH have been described previously (15).

Pediatric SAP diagnosis should meet all of the following requirements: (a) individuals younger than 18 years; (b) the diagnosis of HAdV infection was based on positive nasopharyngeal aspiration (NPA) nucleic acid test or the metagenomic next-generation sequencing (mNGS) (HuaDa, Shenzhen, China); (c) severe CAP was defined according to the standard published by the Pediatric Infectious Diseases Society (PIDS) and the Infectious Diseases Society of America (IDSA) in 2011 (1): when a child had ≥1 major or ≥2 minor criteria: Major criteria: (a) invasive mechanical ventilation; (b) fluid refractory shock; (c) acute need for noninvasive positive pressure ventilation; (d) hypoxemia requiring a fraction of inspired oxygen (FiO_2_) greater than inspired concentration or flow feasible in general care area; Minor criteria: (a) respiratory rate higher than WHO classification for age; (b) apnea; (c) increased work of breathing (e.g., retractions, dyspnea, nasal flaring, grunting); (d) arterial oxygen pressure (PaO_2_)/FiO_2_ ratio <250; (e) multilobar infiltrates; (f) pediatric early warning score (PEWS) score >6; (g) altered mental status; (h) hypotension; (i) presence of effusion; (g) comorbid conditions [e.g., Hemoglobin SS disease (HgbSS), immunosuppression, immunodeficiency]; (k) unexplained metabolic acidosis.

Hemophagocytic lymphohistiocytosis diagnosis was made according to the HLH-2004 diagnostic criteria (16): when a child had ≥5 criteria: (a) ferritin ≥500 μg/L; (b) fever (≥38.5°C), length of fever >7 days; (c) splenomegaly; (d) cytopenias in ≥2 lines (hemoglobin <90 g/L, platelets <100 × 10^9^/L, neutrophils <1.0 × 10^9^/L); (e) hypertriglyceridaemia and/or hypofibrinogenaemia [fasting triglycerides (TG) ≥3 mmol/L, fibrinogen <1.5 g/L]; (f) haemophagocytosis in bone marrow or spleen or lymph nodes; (g) low or absent natural killer cell activity; (h) soluble CD_25_ (soluble interleukin-2 receptor) ≥2,400 U/ml.

The exclusion criteria were: (a) individuals with incomplete medical records; (b) referral to other hospitals and hospital stay <24 h. According to the clinical outcome, all cases were divided into SAP (*n* = 94) and SAP-related HLH (*n* = 30) groups. Our study design was approved by the Ethics Committee of Wuhan Children's Hospital.

### Specimen Testing

Nasopharyngeal aspirations of all cases were obtained within 24 h after admission. Immunofluorescence viral testing was performed to identify HAdV, influenza, parainfluenza virus, and respiratory syncytial virus. Sputum culture was completed to identify bacterial and fungal infections. Additionally, the mNGS was performed to identify the detailed information of pathogenic microorganisms. Among the 124 cases, 106 (85.5%) cases were diagnosed with HAdV-7 infection based on the mNGS. 18(14.5%) cases were diagnosed with HAdV infection based on immunofluorescence viral testing, therefore, the serotypes of HAdV were uncertain. Simultaneously, blood samples were collected and evaluated at the day of admission, 1 week after admission, the point of disease progression, or discharge. All cases underwent laboratory tests including complete blood count, hypersensitive C-reactive protein (Hs-CRP), erythrocyte sedimentation, liver function, myocardial enzyme spectrum, ferritin, coagulation function, serum lipids, inflammatory cytokines, lymphocyte subpopulations, and other tests.

### Treatment Strategy

Once patients were diagnosed with SAP, treatment with intravenous immunoglobulin (IVIG, 1 g/kg for 2 days or 400–500 mg/kg for 4–5days) or glucocorticoid (e.g., methylprednisolone, 1–2 mg/kg for 5–7 days) was administered immediately (17). If the patient is in remission, symptomatic treatment will be continued. Otherwise, further assessments were done promptly. When a child had ≥5 HLH-2004 diagnostic criteria, the diagnosis of HLH was confirmed, and a longer course of glucocorticoid treatment was implemented (16). Meanwhile, Hematology was invited to participate in the medical group for further evaluation and treatment of HLH.

### Data Collection

Clinical data were collected from electronic medical record systems. Demographic characteristics, including age, sex, length of fever, length of wheezing, co-infection, complications, and other general medical histories, were recorded. Laboratory parameters, imaging findings, treatment, and clinical prognosis were obtained from the patient's medical records. It's worth noting that the assessment of clinical data was based on the worst value indicators during hospitalization.

### Statistical Analysis

Statistical analysis was performed using IBM SPSS software (version 22.0, Armonk, NY, United States). The counting data were expressed as the number of cases (*n*) and percentage (%). Chi-square (χ^2^) tests or Fisher's exact tests were used to evaluate the differences in categorical variables. Continuous variables with normal distribution are presented as means ± standard deviation ($\bar{x}\pm s$), and were compared between groups using the dependent-sample *t-*tests. Measurement data of non-normal distribution, expressed as a median and interquartile range [*M* (*P*_25_, *P*_75_)], was checked by Mann-Whitney *U*-tests. Binary logistic regression analyses were performed to evaluate risk factors for the development of HLH in children with SAP. Receiver operator characteristic curves (ROC) and the area under the ROC curve (AUC) were constructed to evaluate the prognostic value of different parameters for the development of HLH in children with SAP. The statistical significance was considered at *p* < 0.05 for two-sided tests.

## Results

### Demographic Features and Clinical Characteristics

**Figure 1:**
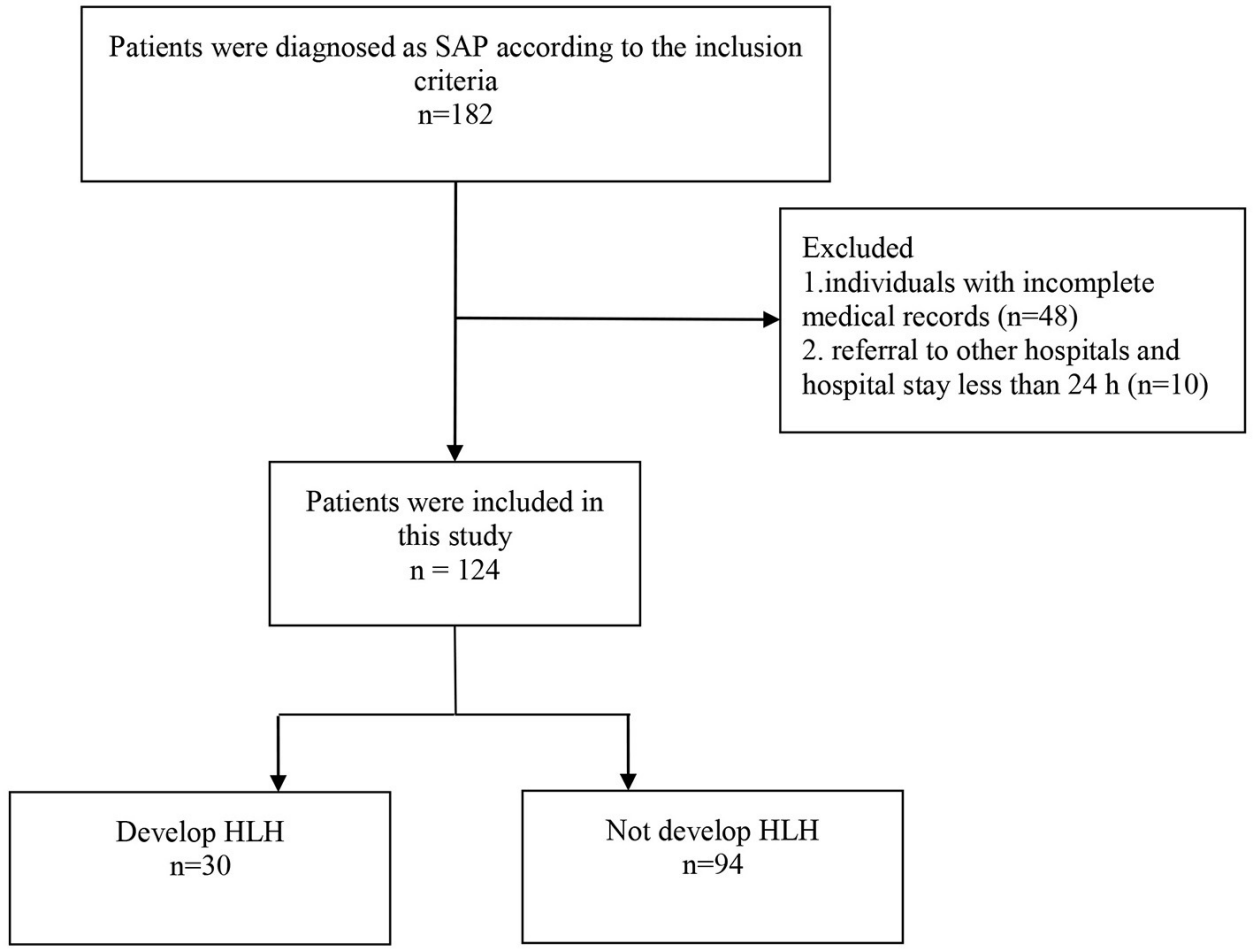
Patients included in the study analysis.

A total of 124 children were diagnosed with SAP, and 30 out of 124 cases were diagnosed with HLH. The recruiting procedure was described in Figure 1. The cases included 83 (66.9%) boys and 41 (33.1%) girls, with a sex ratio of 2.02:1. The median age of the 124 cases was 1.50 years (IQR: 0.89–2.75). Regarding age subgroups, 36 (29.0%) aged <1 year, 64 (51.6%) aged 1–3 years, and 24 (19.4%) aged ≥3 years. The median duration of fever was 11 days (IQR: 9–16), and the median length of hospital stay was 10 days (IQR: 7–15).

Among 124 cases of the study, splenomegaly was detected in 48 cases (38.7%), and 98(79.0%) cases had persistent wheezing over 1 week. Intrapulmonary complications were found in 118 (95.6%) cases. One hundred and seventeen cases (94.4%) had segmental pulmonary consolidation, 106 (85.5%) cases had respiratory failure, 64 cases (51.6%) had pleural effusion, 14 cases (11.3%) had pneumothorax or mediastinal emphysema, and 10 cases (8.1%) had plastic bronchitis. Extrapulmonary complications were found in 102 (82.5%) cases. Eighty-four cases (67.7%) had myocardial damage, 35 cases (28.2%) had liver function damage, 26 cases (20.9%) had septic encephalopathy, and 21 cases (16.9%) had coagulation disorders. Additionally, 52 cases (41.9%) were associated with co-infection. Twenty-eight cases (22.5%) had invasive fungal infections. *Candida albicans*, the most common type of fungal infection, was found in 20 cases (16.1%). Twenty-six cases (20.9%) had mixed bacterial infections. *Haemophilus influenzae*, the most common type of bacterial infection, was found in 18 cases (14.5%).

To prevent the exacerbations of the condition, all 124 cases were treated with IVIG or glucocorticoid. Intravenous immunoglobulin was selected as the first choice in 120 cases (96.8%), and only four cases (3.2%) preferred glucocorticoid. Otherwise, 105 cases (84.7%) with exacerbations were treated with IVIG combined with glucocorticoid. Among the 30 SAP-related HLH cases, the total course of glucocorticoid treatment was 1–2 months. Only one case received chemotherapy according to the 2004-HLH protocol.

In-hospital death occurred in four cases, resulting in a mortality rate of 3.2%. Besides, 33 cases (26.6%) had developed obliterans bronchitis. The detailed clinical characteristics of the patients are presented in Table 1.

**Table 1:** Clinical characteristics of the patients included in this study.

**Characteristic**	**SAP (*n* = 94)**	**SAP-related HLH (*n* = 30)**	***χ^2^/Z/t*-test**	***P***
Male (*n*, %)	63(67.0)	20(66.7)	0.001	0.971
Age [years, *M* (*P_25_, P_75_*)]	1.6(0.9,2.9)	1.2(0.7,1.8)	−2.276	0.023
<1 year (*n*, %)	23(24.5)	13(43.3)		
1–3 years (*n*, %)	49(52.1)	15(50.0)		
≥3 years (*n*, %)	22(23.4)	2(6.7)		
Length of onset-to-treatment time [days, *M* (*P_25_, P_75_*)]	9.0(7.0,14.3)	7.0(6.0,10.0)	−1.633	0.102
Length of fever [days, *M* (*P_25_, P_75_*)]	10.5(9.0,13.0)	17.0(13.8,22.0)	−5.865	0.000
Length of hospital stay [days, *M* (*P_25_, P_75_*)]	9.0(7.0,14.0)	15.5(13.0,27.5)	−5.227	0.000
Splenomegaly (*n*, %)	32(34.0)	16(53.3)	3.567	0.059
Persistent wheezing (*n*, %)	72(76.6)	26(86.7)	1.392	0.238
Laboratory findings				
TG [mmol/L, *M* (*P_25_, P_75_*)]	1.99(1.45,2.51)	3.74(3.46,4.44)	−6.894	0.000
Ferritin [ng/ml, *M* (*P_25_, P_75_*)]	906.55(376.08,1483.83)	3226.95(1805.83,7683.30)	−6.290	0.000
Albumin (g/L, $\bar{x}\pm s$)	35.64 ± 5.26	27.27 ± 3.93	0.013	0.000
Hs-CRP [mg/L, *M* (*P_25_, P_75_*)]	21.85(10.41,43.33)	39.70(13.48,77.83)	−2.004	0.045
CD3 (%,$\bar{x}\pm s$)	53.02 ± 11.93	52.25 ± 11.22	0.312	0.755
CD19 (%,$\bar{x}\pm s$)	36.22 ± 12.44	37.71 ± 13.35	−0.563	0.575
NK [%, *M* (*P_25_, P_75_*)]	7.06(4.10,11.91)	4.41(3.11,9.45)	−1.841	0.066
CD4/CD8	1.39(1.02,1.93)	1.27(1.01,1.88)	−0.662	0.508
IL-2 [pg/ml, *M* (*P_25_, P_75_*)]	2.00(1.62,2.41)	2.26(1.70,2.78)	−1.567	0.117
IL-4 [pg/ml, *M* (*P_25_, P_75_*)]	2.22(1.96,3.21)	2.94(2.09,4.45)	−1.713	0.087
IL-6 [pg/ml, *M* (*P_25_, P_75_*)]	99.96(37.89,238.72)	336.69(40.08,520.26)	−2.845	0.004
IL-10 [pg/ml, *M* (*P_25_, P_75_*)]	22.18(11.72,34.31)	30.56(20.36,59.43)	−2.249	0.024
TNF-α [pg/ml, *M* (*P_25_, P_75_*)]	2.16(1.24,3.00)	2.13(1.66,2.85)	−0.315	0.753
INF-γ [pg/ml, *M* (*P_25_, P_75_*)]	51.08(20.23,102.42)	51.94(26.69,132.92)	−0.592	0.554
HAdV-7 infection (*n*, %)	82(87.2)	24(80.0)	0.959	0.327
**Imaging findings (** ***n*** **, %)**				
Segmental pulmonary consolidation	88(93.6)	29(96.7)	0.397	0.529
Pneumothorax or mediastinal emphysema	8(8.5)	6(20.0)	2.997	0.083
Pleural effusion	46(48.9)	18(60.0)	1.115	0.291
**Co-infection (** ***n*** **, %)**				
*H. influenzae*	14(14.9)	4(13.3)	0.045	0.833
Invasive fungal infection	18(19.1)	10(33.3)	2.617	0.106
**Complications (** ***n*** **, %)**				
Respiratory failure	78(83.0)	28(93.3)	1.965	0.161
Plastic bronchitis	6(6.4)	4(13.3)	1.482	0.223
Myocardial damage	62(66.0)	22(73.3)	0.566	0.452
Hepatic dysfunction	23(24.5)	12(40.0)	1.654	0.198
Toxic encephalopathy	17(18.1)	9(30.0)	1.948	0.163
Coagulation disorders	14(14.9)	7(23.3)	1.152	0.283
**Treatment (** ***n*** **, %)**				
IVIG combined with glucocorticoids	78(83.0)	27(90.0)	0.864	0.353
IVIG-alone	14(14.9)	1(3.3)	2.858	0.091
Glucocorticoids-alone	2(2.1)	2(6.7)	1.501	0.221
**Outcomes**				
Mortality (*n*, %)	1(1.1)	3(10.0)	5.817	0.016
Obliterans bronchitis	18(19.1)	15(50.0)	11.083	0.001

*HAdV, human adenovirus; TG, triglycerides; Hs-CRP, hypersensitive C-reactive protein; CD, cluster of differentiation; NK, natural killer cell; IL, interleukin; TNF-α, tumor necrosis factor α; INF-γ, interferon γ; H. influenzae, Haemophilus influenzae*.

### Risk Factors for Patients With SAP to Develop HLH

As shown in Table 2, univariate analysis identified age (*OR* = 0.635, 95%*CI*: 0.418–0.965, *p* = 0.033), length of fever (*OR* = 1.299, 95%*CI*: 1.170–1.441, *p* = 0.000), TG (*OR* = 9.669, 95%*CI*: 4.084–22.891, *p* = 0.000), ferritin (*OR* = 1.001, 95%*CI*: 1.000–1.001, *p* = 0.000), albumin (*OR* = 0.682, 95%*CI*: 0.588–0.791, *p* = 0.000), Hs-CRP (*OR* = 1.016, 95%*CI*: 1.003–1.030, *p* = 0.020), IL-6 (*OR* = 1.003, 95%*CI*: 1.001–1.005, *p* = 0.001), and IL-10 (*OR* = 1.015, 95%*CI*: 1.000–1.030, *p* = 0.049) as potential risk factors for SAP to develop HLH. The results of the multivariate analysis confirmed that the length of fever (*OR* = 1.331, 95%*CI*: 1.002–1.769, *p* = 0.048), and TG (*OR* = 17.345, 95%*CI*: 1.358–221.538, *p* = 0.028) were independent risk factors for SAP developing HLH.

**Table 2:** Logistic regression analysis of risk factors for SAP to develop HLH.

**Factors**	**Univariate analysis**		**Multivariate analysis**	
	***p***	***OR* (95% *CI*)**	***p***	***OR* (95% *CI)***
Age	0.033	0.635 (0.418, 0.965)	0.635	1.255 (0.491, 3.205)
Length of fever	0.000	1.299 (1.170, 1.441)	0.048	1.331 (1.002, 1.769)
TG	0.000	9.669 (4.084, 22.891)	0.028	17.345 (1.358, 221.538)
ferritin	0.000	1.001 (1.000, 1.001)	0.153	1.001 (1.000, 1.001)
Albumin	0.000	0.682 (0.588, 0.791)	0.103	0.807 (0.624, 1.044)
Hs-CRP	0.020	1.016 (1.003, 1.030)	0.447	1.015 (0.977, 1.054)
IL-6	0.001	1.003 (1.001, 1.005)	0.092	1.005 (0.999, 1.012)
IL-10	0.049	1.015 (1.000, 1.030)	0.481	1.016 (0.972, 1.061)

*SAP, severe adenovirus pneumonia; HLH, hemophagocytic lymphohistiocytosis; OR, odds ratio; CI, confidence interval; TG, triglycerides; Hs-CRP, hypersensitive C-reactive protein; IL, interleukin*.

### The Value of Risk Factors for Patients With SAP to Develop HLH

**Figure 2:**
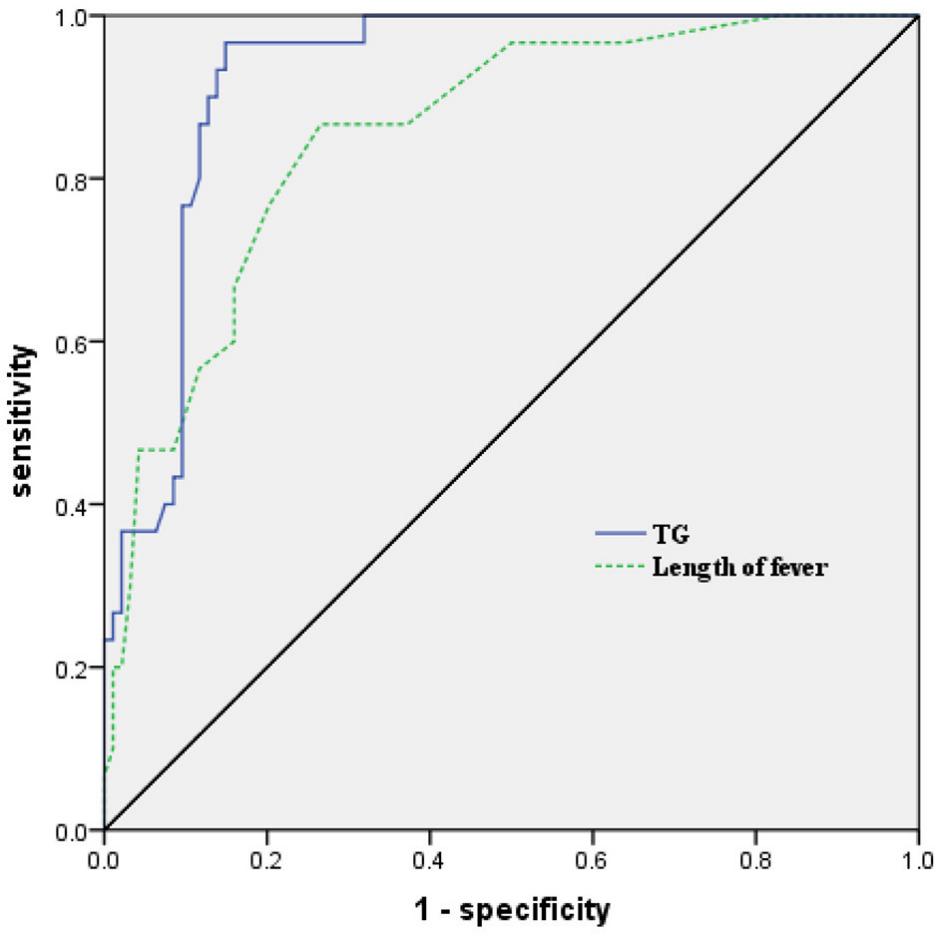
Receiver operating characteristic (ROC) curve analysis of TG and the length of fever in predicting the development of HLH in children with SAP. TG, triglycerides; HLH, hemophagocytic lymphohistiocytosis; SAP, severe adenovirus pneumonia.

To further examine the predictive value of the above factors, the prediction model was examined by ROC curve analysis. The AUC of TG in the prediction of HLH was 0.924 (95%*CI*: 0.879–0.970, *P* < 0.001), and the optimal cut-off point of TG in the prediction of HLH was 3.02 mmol/L, with a sensitivity of 96.7% and a specificity of 85.1%. The AUC of the length of fever in the prediction of HLH was 0.855(95%*CI*: 0.781–0.928, *P* < 0.001), and the optimal cut-off point of the length of fever in the prediction of HLH was 12.5 days, with a sensitivity of 86.7% and a specificity of 73.4% (Figure 2).

## Discussion

To the best of our knowledge, the present study was the first to investigate the risk factors for the development of HLH in children with SAP. Our study suggested that two factors were associated with the development of HLH, which were the length of fever (*OR* = 1.331, 95%*CI*: 1.002–1.769) and TG (*OR* = 17.345, 95%*CI*: 1.358–221.538).

Human adenovirus, a non-enveloped double-stranded DNA virus, is one of the most common viruses responsible for viral respiratory diseases in children (4). Human adenovirus is more common among children younger than 3 years old, and it occurs frequently in the winter and spring seasons (4, 18). Fever, cough, sore throat, and conjunctival congestion are the most common symptoms of mild HAdV infections. However, once a child presented with prolonged fever and cough for more than 7 days, the possibility of SAP should be considered. Severe HAdV infections can lead to serious multiple viscera damages, especially for HLH (11, 12, 14, 19). The previous studies showed that HAdV-7 causes a more severe disease than the other serotype, with a higher intensive care unit admission rate and a longer length of hospital stay (20). The underlying mechanism may be related to a higher viral load and inflammatory response (5, 21, 22). In the present study, the median age of all cases was 1.50 years, with 100 cases (80.6%) younger than 3 years old. The median duration of fever was 11 days, and the median length of hospital stay was 10 days. The HAdV-7 was the most common serotype in hospitalized children, with a proportion of 85.5%. These results were similar to the findings of previous studies (5, 18, 20, 22). It's reported that SAP progressed with multiple organ dysfunction syndromes, the mortality is higher over 50% (23). In our study, the in-hospital death occurred in four cases, resulting in a mortality rate of 3.2%. Furthermore, there was only 10% of in-hospital mortality even in the SAP-related HLH group. We speculate that the reason for this difference resides in two aspects. On the one hand, this remarkable difference could be due to individual differences between different study populations. On the other hand, an aggressive early treatment might improve the prognosis of some patients. The current clinical treatments for SAP are mainly symptomatic treatments and the prevention of multiple viscera complications. Oxygen inhalation, mechanical ventilation, IVIG, glucocorticoids, and plasmapheresis are viable treatments for patients with SAP. However, even after the intervention, some children still had developed HLH. Hence, early recognition and avoiding natural progression from early stages should be the main objective to fight against SAP in children. In our study, we assessed the risk factors for patients with SAP to develop HLH using the quantitative method. The results demonstrated that it is more likely to develop HLH when the length of fever is longer than 12.5 days and the level of TG is more than 3.02 mmol/L.

Hemophagocytic lymphohistiocytosis, an uncommon blood disorder in children, is due to the proliferation and activation of macrophages in response to a cytokine storm. Aside from genetic factors, inflammatory diseases are prominent in the development of HLH. Epstein-Barr viral infection is the most common infectious trigger of acquired HLH, accounting for about 90% (10). Besides, mycoplasma, fungus, and bacteria can also lead to HLH (16, 24). Hemophagocytic lymphohistiocytosis is a rare complication of HAdV infection, and the pathogenesis of HAdV-induced HLH remains unknown (11–14). It has been hypothesized that immunologic activation and dysregulation are involved in the pathophysiological processes. Innate immune activation is critical to control infections and acts as a bridge for adaptive immunity. However, innate immune overactivation is detrimental in multiple viscera functions. Human adenovirus infection can result in overactivation of the innate immune system, and it induces innate immune cells (macrophage, NK cell, and neutrophil) to release a large number of cytokines, like IL-1, IL-2, IL-6, IL-12, IFN-α, IFN-β, and IFN-γ, which in turn can lead to inflammatory dysregulation and cytokine storm (25). Meanwhile, HAdV infection results in dysfunction of NK cell, which renders the NK cell inefficient at clearing virus. Furthermore, the complement system, an important component in innate immunity, can be initiated through three main pathways. Although the pathways are triggered independently, all of the complement cascades culminate in the cleavage of C3 to C3a and C3b. Human adenovirus infection can readily initiate amplification of the activation cascades, and lead to a sustained high plasma level of C3a (5, 26). Taken together, all of these changes lead to the perpetuation of the inflammatory response, and the development of HLH in children with SAP (27, 28).

Clinically, HAdV infection mainly involves the respiratory system in the early stage of the disease (2, 22). As the disease progresses, inflammatory dysregulation and cytokine storm can lead to HLH, including but not limited to persistent fever, hepatosplenomegaly, cytopenia, hypertriglyceridemia, and hypofibrinogenaemia (13, 14). The treatment of HAdV-induced HLH remains controversial. Some scholars hold the view that aggressive treatment of the primary disease can improve the prognosis of acquired HLH (16). However, it seems necessary to actively screen for familial HLH genes in cases with non-response to treatment. It can be also hypothesized that switching cases to a more intensive treatment regimen based on familial HLH gene-positive findings might have resulted in more favorable outcomes (10, 16, 29).

## Study Limitations

The present study has several limitations. First, the genetic test was not covered by the national health insurance in China. Therefore, only a few poor prognosis cases were tested for familial HLH-associated genes, and the missed diagnoses could not be excluded. Second, the study was a single-center retrospective study, which could introduce observation bias. Last, the number of patients involved was relatively small. Thus, more multi-center prospective studies are needed to identify the conclusions in our study.

## Conclusions

In conclusion, early recognition and avoiding natural progression from early stages should be the main objective to fight against HAdV-induced SAP in children. Children with SAP who had a duration of fever over 12.5 days and the TG level over 3.02 mmol/L are more likely to develop HLH.

## Data Availability Statement

The original contributions presented in the study are included in the article/supplementary material, further inquiries can be directed to the corresponding author/s.

## Ethics Statement

The studies involving human participants were reviewed and approved by Ethics Committee of Wuhan Children's Hospital. Written informed consent to participate in this study was provided by the participants' legal guardian/next of kin. Written informed consent was obtained from the individual(s), and minor(s)' legal guardian/next of kin, for the publication of any potentially identifiable images or data included in this article.

## Author Contributions

H-yZ, MX, and YZ designed and conceived the experiments. H-yZ, MX, FY, and M-rZ performed the experiments. H-yZ, MX, and FY analyzed the data. H-yZ and MX contributed to the reagents, materials, and analysis tools. H-yZ and MX wrote the manuscript. All authors contributed to the article and approved the submitted version.

## Conflict of Interest

The authors declare that the research was conducted in the absence of any commercial or financial relationships that could be construed as a potential conflict of interest.

## Publisher's Note

All claims expressed in this article are solely those of the authors and do not necessarily represent those of their affiliated organizations, or those of the publisher, the editors and the reviewers. Any product that may be evaluated in this article, or claim that may be made by its manufacturer, is not guaranteed or endorsed by the publisher.

## References

[1] Bradley JS Byington CL Shah SS Alverson B Carter ER Harrison C. The management of community-acquired pneumonia in infants and children older than 3 months of age: clinical practice guidelines by the Pediatric Infectious Diseases Society and the Infectious Diseases Society of America. Clin Infect Dis. (2011) 53:e25–76. 10.1093/cid/cir531 21880587 PMC7107838

[2] Chen HL Chiou SS Hsiao HP Ke GM Lin YC Lin KH. Respiratory adenoviral infections in children: a study of hospitalized cases in southern Taiwan in 2001–2002. J Trop Pediatr. (2004) 50:279–84. 10.1093/tropej/50.5.279 15510759

[3] Yun BY Kim MR Park JY Choi EH Lee HJ Yun CK. Viral etiology and epidemiology of acute lower respiratory tract infections in Korean children. Pediatr Infect Dis J. (1995) 14:1054–9. 10.1097/00006454-199512000-00005 8745017

[4] Ma J Duffy MR Deng L Dakin RS Uil T Custers J. Manipulating adenovirus hexon hypervariable loops dictates immune neutralisation and coagulation factor X-dependent cell interaction in vitro and in vivo. PLoS Pathog. (2015) 11:e1004673. 10.1371/journal.ppat.1004673 25658827 PMC4450073

[5] Fu Y Tang Z Ye Z Mo S Tian X Ni K. Human adenovirus type 7 infection causes a more severe disease than type 3. BMC Infect Dis. (2019) 19:36. 10.1186/s12879-018-3651-2 30626350 PMC6327436

[6] Wo Y Lu QB Huang DD Li XK Guo CT Wang HY. Epidemical features of HAdV-3 and HAdV-7 in pediatric pneumonia in Chongqing, China. Arch Virol. (2015) 160:633–8. 10.1007/s00705-014-2308-8 25504360 PMC7087000

[7] Ou ZY Zeng QY Wang FH Xia HM Lu JP Xia JQ. Retrospective study of adenovirus in autopsied pulmonary tissue of pediatric fatal pneumonia in South China. BMC Infect Dis. (2008) 8:122. 10.1186/1471-2334-8-122 18803877 PMC2566980

[8] Hartz B Marsh R Rao K Henter JI Jordan M Filipovich L. The minimum required level of donor chimerism in hereditary hemophagocytic lymphohistiocytosis. Blood. (2016) 127:3281–90. 10.1182/blood-2015-12-684498 27099148 PMC5291300

[9] Al-Samkari H Berliner N. Hemophagocytic Lymphohistiocytosis. Annu Rev Pathol. (2018) 13:27–49. 10.1146/annurev-pathol-020117-043625 28934563

[10] Ishii E Ohga S Imashuku S Yasukawa M Tsuda H Miura I. Nationwide survey of hemophagocytic lymphohistiocytosis in Japan. Int J Hematol. (2007) 86:58–65. 10.1532/IJH97.07012 17675268

[11] Censoplano N Gorga S Waldeck K Stillwell T Rabah-Hammad R Flori H. Neonatal adenovirus infection complicated by hemophagocytic lymphohistiocytosis syndrome. Pediatrics. (2018) 141:S475–80. 10.1542/peds.2017-2061 29610175

[12] Mellon G Henry B Aoun O Boutolleau D Laparra A Mayaux J. Adenovirus related lymphohistiocytic hemophagocytosis: case report and literature review. J Clin Virol. (2016) 78:53–6. 10.1016/j.jcv.2016.03.011 26985594

[13] Otto WR Behrens EM Teachey DT Lamson DM Barrett DM Bassiri H. Human adenovirus 7-associated hemophagocytic lymphohistiocytosis-like illness: clinical and virological characteristics in a cluster of five pediatric cases. Clin Infect Dis. (2020) 2020:ciaa1277. 10.1093/cid/ciaa1277 32866230 PMC8492126

[14] La Fay C Bosdure E Baravalle-Einaudi M Stremler-Le Bel N Dubus JC Mazenq J. Severe adenovirus pneumonia with hemophagocytic syndrome and respiratory failure. Arch Pediatr. (2020) 27:383–5. 10.1016/j.arcped.2020.07.003 32811705

[15] Zhang HY Li CJ Long Y Sun DM Wang RG Zhang Y. Clinical features of children with severe adenovirus pneumonia and hemophagocytic syndrome: an analysis of 30 cases. Zhongguo Dang Dai Er Ke Za Zhi. (2020) 22:744–8. 10.7499/j.issn.1008-8830.2003080 32669172 PMC7389625

[16] Henter JI Horne A Arico M Egeler RM Filipovich AH Imashuku S. HLH-2004: diagnostic and therapeutic guidelines for hemophagocytic lymphohistiocytosis. Pediatr Blood Cancer. (2007) 48:124–31. 10.1002/pbc.21039 16937360

[17] National Health Commission of the People's Republic of China State Administration of Traditional Chinese Medicine. Guideline for diagnosis and treatment of adenovirus pneumonia in children (2019 version). Chin J Clin Infect Dis. (2019)12:161–6. 10.3760/cma.j.issn.1674-2397.2019.03.001

[18] Jain S Williams DJ Arnold SR Ampofo K Bramley AM Reed C. Community-acquired pneumonia requiring hospitalization among US children. N Engl J Med. (2015) 372:835–45. 10.1056/NEJMoa1405870 25714161 PMC4697461

[19] Jhanji V Chan TC Li EY Agarwal K Vajpayee RB. Adenoviral keratoconjunctivitis. Surv Ophthalmol. (2015) 60:435–43. 10.1016/j.survophthal.2015.04.001 26077630

[20] Xie L Zhang B Xiao N Zhang F Zhao X Liu Q. Epidemiology of human adenovirus infection in children hospitalized with lower respiratory tract infections in Hunan, China. J Med Virol. (2019) 91:392–400. 10.1002/jmv.25333 30286268 PMC7159165

[21] Xu N Chen P Wang Y. Evaluation of risk factors for exacerbations in children with adenoviral pneumonia. Biomed Res Int. (2020) 2020:4878635. 10.1155/2020/4878635 32802848 PMC7415082

[22] Lai CY Lee CJ Lu CY Lee PI Shao PL Wu ET. Adenovirus serotype 3 and 7 infection with acute respiratory failure in children in Taiwan, 2010-2011. PLoS ONE. (2013) 8:e53614. 10.1371/journal.pone.0053614 23326469 PMC3542335

[23] Chowdhury F Shahid ASMSB Ghosh PK Rahman M Hassan MZ Akhtar Z. Viral etiology of pneumonia among severely malnourished under-five children in an urban hospital, Bangladesh. PLoS ONE. (2020) 15:e0228329. 10.1371/journal.pone.0228329 32017782 PMC6999894

[24] Gu JL Lu ZW Wang WJ Zheng YJ Li J Shao YB. 11 cases of Mycoplasma pneumoniae-associated hemophagocytic syndrome: a case series report. Chin J Evid Based Pediatr. (2020) 15:229–32. 10.3969/j.issn.1673-5501.2020.03.014

[25] Atasheva S Shayakhmetov DM. Adenovirus sensing by the immune system. Curr Opin Virol. (2016) 21:109–13. 10.1016/j.coviro.2016.08.017 27639089 PMC5138075

[26] Tian J Xu Z Smith JS Hofherr SE Barry MA Byrnes AP. Adenovirus activates complement by distinctly different mechanisms *in vitro* and *in vivo*: indirect complement activation by virions *in vivo*. J Virol. (2009) 83:5648–58. 10.1128/JVI.00082-09 19321608 PMC2681959

[27] Janka GE. Hemophagocytic syndromes. Blood Rev. (2007) 21:245–53. 10.1016/j.blre.2007.05.001 17590250

[28] Jordan MB Allen CE Weitzman S Filipovich AH McClain KL. How I treat hemophagocytic lymphohistiocytosis. Blood. (2011) 118:4041–52. 10.1182/blood-2011-03-278127 21828139 PMC3204727

[29] Stepp SE Dufourcq-Lagelouse R Le Deist F Bhawan S Certain S Mathew PA. Perforin gene defects in familial hemophagocytic lymphohistiocytosis. Science. (1999) 286:1957–9. 10.1126/science.286.5446.1957 10583959

